# A D-Shaped Polymer Optical Fiber Surface Plasmon Resonance Biosensor for Breast Cancer Detection Applications

**DOI:** 10.3390/bios14010015

**Published:** 2023-12-28

**Authors:** Xun Wu, Ying Wang, Jiaxiong Zhang, Yunfang Zhang, Xing Rao, Chen Chen, Han Liu, Yubin Deng, Changrui Liao, Mateusz Jakub Smietana, George Yuhui Chen, Liwei Liu, Junle Qu, Yiping Wang

**Affiliations:** 1 Shenzhen Key Laboratory of Photonic Devices and Sensing Systems for Internet of Things, Guangdong and Hong Kong Joint Research Centre for Optical Fibre Sensors, State Key Laboratory of Radio Frequency Heterogeneous Integration, Shenzhen University, Shenzhen 518060, China; 2 Shenzhen Key Laboratory of Ultrafast Laser Micro/Nano Manufacturing, Key Laboratory of Optoelectronic Devices and Systems of Ministry of Education/Guangdong Province, College of Physics and Optoelectronic Engineering, Shenzhen University, Shenzhen 518060, China; 3 Guangdong Laboratory of Artificial Intelligence and Digital Economy (SZ), Shenzhen 518107, China; 4 Division of Microsystem & Electronic Materials Technology, Institute of Microelectronics & Optoelectronics, Warsaw University of Technology, Koszykowa 75, 00-662 Warsaw, Poland

**Keywords:** polymer optical fiber, surface plasmon resonance, breast cancer detection, fiber sensor

## Abstract

Fiber-optic biosensors have garnered significant attention and witnessed rapid development in recent years owing to their remarkable attributes such as high sensitivity, immunity to electromagnetic interference, and real-time monitoring. They have emerged as a potential tool in the realm of biomarker detection for low-concentration and small molecules. In this paper, a portable and cost-effective optical fiber biosensor based on surface plasmon resonance for the early detection of breast cancer is demonstrated. By utilizing the aptamer human epidermal growth factor receptor 2 (HER2) as a specific biomarker for breast cancer, the presence of the HER2 protein can be detected through an antigen-antibody binding technique. The detection method was accomplished by modifying a layer of HER2 aptamer on the flat surface of a gold-coated D-shaped polymer optical fiber (core/cladding diameter 120/490 μm), of which the residual thickness after side-polishing was about 245 μm, the thickness of the coated gold layer was 50 nm, and the initial wavelength in pure water was around 1200 nm. For low-concentration detection of the HER2 protein, the device exhibited a wavelength shift of ~1.37 nm with a concentration of 1 μg/mL (e.g., 5.5 nM), which corresponded to a limit of detection of ~5.28 nM. Notably, the response time of the biosensor was measured to be as fast as 5 s. The proposed biosensor exhibits the potential for early detection of HER2 protein in initial cancer serum and offers a pathway to early prevention of breast cancer.

## 1. Introduction

Breast cancer holds the highest incidence rate among female malignant tumors worldwide, affecting approximately 24.2% of women [1]. The initial stages of breast cancer often exhibit subtle symptoms, while advanced stages can lead to the development of distant metastases and widespread organ lesions, posing a direct threat to the patient’s life. Within breast cancer cells, three markers—estrogen receptor (ER), progesterone receptor (PR), and human epidermal growth factor receptor 2 (HER2)—are often used to determine the existence and concentration of cancer cells. Among them, ER and PR are located inside the cell and HER2 is situated on the cell surface. As a member of the human epidermal growth factor receptor (EGFR) family, HER2 plays a crucial role in the development of invasive breast cancer through its overexpression [2]. In human breast cancer cell lines, this gene is frequently amplified, and the expression of HER2 has been found to be twice as high in breast cancer patients without bone metastases compared to those with bone metastases [3]. Early detection, diagnosis, and treatment of breast cancer through population screening have the potential to significantly improve survival rates, underscoring the importance of achieving the accurate detection of breast cancer markers in humans [4]. Meanwhile, HER2 gene amplification and receptor overexpression, observed in approximately 15% to 20% of breast cancer patients, also serve as significant prognostic markers associated with poor outcomes, including more aggressive disease and shorter survival [5]. Moreover, HER2-positive status is considered a predictive marker for the response to targeted drugs that specifically act on HER2, such as trastuzumab and lapatinib [6,7].

At present, various methods have been developed to detect HER2 marker site parameters and cancer cells [8], including electrochemical detection [9], fiber-optic devices [10], acoustic sensors [11], quartz crystal microbalance [12], field-effect transistors [13], and semiconductor quantum dots [14]. Compared to these methods, fiber-optic sensing technology, especially fiber-optic surface plasmon resonance (SPR) biosensing, has emerged as a promising solution for HER2 protein detection in recent years due to the advantages of user-friendly operation and the capability for remote and real-time detection. Fiber-optic SPR sensors, developed by Jorgenson and Yee, employ a fiber-optic design that replaces the traditional prism-based system, which enables the adoption of SPR biosensing outside of research centers due to the limitations of high cost and dedicated operation; thus, they have gained widespread attention in the detection of bioconjugation reactions [15,16,17]. After the first demonstration of a fiber SPR device, Liedberg et al. applied the fiber SPR technology to biochemical detection [18], since biomass measurement can be achieved by changes in biomass that affect the refractive index (RI) near the surface of the sensing structure [19]. Wong et al. proposed a gold-coated photonic crystal fiber (PCF) SPR sensor to monitor the binding kinetics of human immunoglobulin G (IgG) and obtained a detection limit (DL) of 0.267 mg/L [20]. In 2016, Zhao et al. proposed a D-shaped optical fiber SPR probe for avian influenza virus sensing, achieving a DL of up to 5.14 × 10^5^ EID50/0.1 mL and an averaged response time of 10 min [21]. In 2019, Wang et al. proposed a highly sensitive fiber SPR biosensor based on graphene oxide (GO) and staphylococcal protein A (SPA) co-modified tilted fiber Bragg grating (TFBG) to detect IgG, exhibiting a sensitivity 0.096 dB/(μg/mL) and a DL of 0.5 μg/mL [22]. Recently, Zhang et al. achieved temperature-compensated acetylcholine-specific measurement based on a U-fiber SPR biosensor with a DL of 30 nM [23]. However, most of the fiber SPR sensors reported so far are made of silica fiber, of which the material RI is much higher than that of water and, thus, the sensitivity is limited to approximately 2000 nm/RIU for biosensing in human blood solutions [24]. To enhance the sensitivity of fiber-optic SPR sensors, technologies including long-range SPR [25], PCFs [26], polymer optical fibers (POFs) [27], two-dimensional materials [28], and metamaterials [29] have been investigated broadly. Among them, the utilization of a low-RI POF exhibits extremely significant sensitivity improvement, since the sensitivity of SPR sensors is most sensitive when the RI of the analyte closely matches the RI of the fiber substrate material, as has been demonstrated previously [30].

Building upon this insight, we employed a low-RI polymer optical fiber to develop a highly sensitive SPR biosensor for breast cancer detection applications [31]. The fiber used herein is a commercially available CYTOP polymer optical fiber that initially was intended for application in short-range optical data transmission and exhibits excellent mechanical toughness sufficient to be side-polished to a D-shape [32]. With a coating of gold film 50 nm thick onto the polished flat surface of the D-shaped polymer optical fiber, the obtained fiber SPR device exhibited an RI sensitivity of 28,100 nm/RIU within the range of 1.330–1.335, which is ten times higher than that of conventional silica fiber SPR sensors [33]. For the detection of the HER2 protein, a layer of HER2 aptamer was modified onto the gold layer of the fabricated fiber SPR device to achieve the antigen–antibody binding. The device exhibited a detection limit of ~5.28 nM and a response time of ~5 s for low-concentration HER2 protein detection. The proposed fiber-optic SPR sensor represents a helpful attempt to achieve a high-sensitivity and cost-effective method of detection of breast cancer, and is expected to be a promising tool for applications in the fields of biosensing and bioanalysis.

## 2. Biosensor Design and Fabrication

### 2.1. Simulation

**Figure 1:**
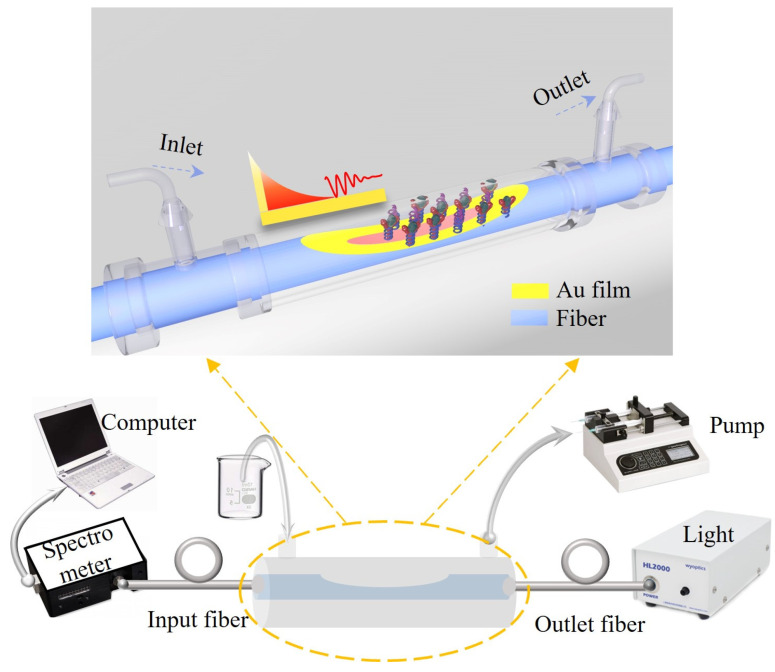
Schematic diagram of the proposed POF SPR biosensor and the experimental setup for bio-modification and bio-detection. Inset shows the sensing area of the fiber-optic SPR biosensor sealed in a microfluidic chip.

Figure 1 shows the schematic diagram of the proposed POF fiber SPR biosensor and corresponding experimental setup for the microfluidic detection of HER2 proteins. The proposed biosensor employs the well-known D-shaped fiber structure, which can be obtained by polishing off one half of the original cylindrical optical fiber [34]. After coating a thin layer of gold film onto the flat surface of the D-shape region, SPR can be excited at the metal interface through the evanescent field of the fiber core mode. To investigate the relationship between the gold film’s thickness and the device’s sensitivity, full width at half maximum (FWHM), and figure of merit (FOM), a finite element model was established according to the geometry of the D-shape POF fiber, of which the core/cladding diameter and residual thickness were 50/490 μm and 245 μm, respectively. The material RI of the fiber core/cladding was 1.357/1.342 (nominated at 589 nm), taking into account the material dispersion in the simulation. And the thickness of the gold film was changed from 30 to 70 nm, with a step of 10 nm. 

**Figure 2:**
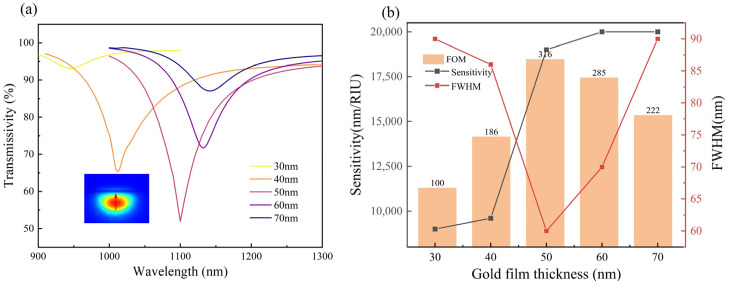
(**a**) Transmission spectra of sensors with different gold film thicknesses at 1.330 refractive index, inset shows the mode field distribution at resonant wavelengths. (**b**) Sensitivity, half-wavelength, and figure of merit of d-shape plastic SPR fiber sensors with different gold film thicknesses.

The simulated transmission spectra, sensitivity, FWHM, and FOM of the proposed POF SPR device are plotted in Figure 2. Under an external RI of 1.330, the resonant wavelength gradually redshifted from 945 to 1130 nm, with the gold film’s thickness increasing from 30 to 70 nm. The resonance depth gradually approached 50% (theoretical limit) when the gold film thickened to 50 nm, and then decays with further thickening of the gold film, as can be seen clearly in Figure 2a. Notably, the resonant depth at a film thickness of 30 or 70 nm was less than 1/4 of that at a film thickness of 50 nm, indicating that both extremely thin and thick gold films yield insufficient coupling for the proposed SPR sensor [21]. Figure 2b illustrates the plots of sensor sensitivity and FWHM, as well as the bar charts for FOM, corresponding to various gold film thicknesses at an external RI of 1.330. As depicted, the sensitivity progressively increased from 6000 to 20,000 nm/RIU, with the gold film’s thickness increasing from 30 to 70 nm. Besides the sensitivity, the detection limit of the proposed sensor was also limited by the FWHM. A more comprehensive evaluation is FOM, which is defined as the ratio of sensitivity to FWHM. The maximum FOM obtained in the simulation was ~316.7, corresponding to a gold film thickness of 50 nm. Therefore, the optimal thickness of the gold film was determined to be 50 nm for the proposed D-shaped polymer fiber SPR sensor.

### 2.2. Device Fabrication

**Figure 3:**
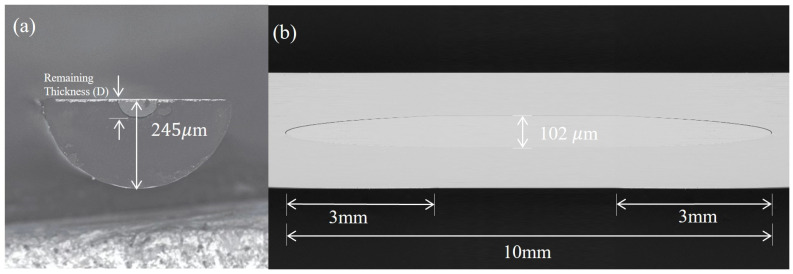
(**a**) SEM image of the D-shaped polymer optical fiber. (**b**) Side SEM image of D-shaped polymer fiber; flat area length is 4 mm.

The D-shaped SPR sensor was fabricated by a commercially available low-RI POF (GigaPOF-50SR, Chromis Fiber, NJ, USA) with a core/cladding diameter of 50/490 μm, which was made up of perfluorinated optical polymer (CYTOP). To prepare the D-shaped fiber, the polymer fiber was polished by the side-polishing technique which we developed and that has been reported elsewhere [33,34]. During the polishing process, the polymer fiber was securely placed under a computer-controlled polishing wheel equipped with sandpaper. Initially, the polishing length was set at 5 mm, and coarse-grade sandpaper was used to reduce the fiber’s thickness to approximately 325 μm. Then, a fine polishing process was performed using 5000 grit sandpaper to achieve a thickness of about 250 μm. Note that the polishing surface was near the fiber core at this point. Subsequently, the fiber was polished more precisely with 10,000 grit sandpaper to achieve a remaining thickness of 245 μm, the scanning electron microscopy (SEM) image of which, in cross-sectional view, is depicted in Figure 3a. After the side-polishing process, the polished area of the D-shaped fiber was immersed in a piranha solution that consisted of sulfuric acid and hydrogen peroxide in a 3:1 ratio for 30 min. Then, the sample was thoroughly cleaned with ethanol. Finally, a gold film 50 nm thick was deposited onto the D-shaped fiber through the vacuum magnetron sputtering technique. Figure 3b shows the SEM image of the polished region after gold film coating. It should be noted that the image is spliced and scaled along the direction of the fiber’s length for full display.

### 2.3. Bio-Modification

**Figure 4:**
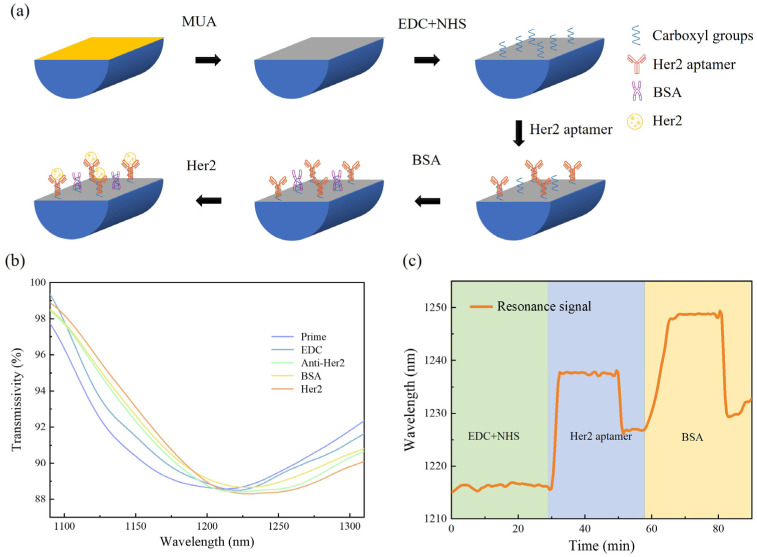
(**a**) Flow chart of surface modification of SPR sensing area of D-shaped fiber. (**b**) Spectral intensity changes during modification. (**c**) Wavelength shifts during biomodification.

Before HER2 detection, HER2 antibodies should be fixed onto the flat gold surface of the polymer fiber SPR sensor, which can be accomplished through bio-modification processes with the aid of microfluidic techniques. The experimental setup is shown in Figure 1, where two polypropylene T-joints with inner diameters of 0.9 mm are utilized as the inlet and outlet of liquid samples, respectively. The polymer fiber SPR sensor is threaded through the horizontal microfluidic channel that connected to the inlet and outlet T-joints. Both ends of the horizontal microfluidic channel are secured using UV curable adhesive to prevent air from entering the system. Liquids can be pumped into the microfluidic channel through the inlet with a microfluidic syringe pump. After passing through the D-shaped sensing area of the SPR sensor, the liquid sample exits through the outlet. To monitor the SPR wavelength in real time, one end of the polymer fiber SPR sensor is connected to a halogen lamp, and the other end is connected to a computer-controlled spectrometer (NIRQuest 512, (NIRQuest512, Ocean Optics, Shanghai, China). Figure 4a shows the bio-modification flowchart of the sensing area of the D-shaped polymer fiber sensor. To prepare the gold film surface for bio-conjugation, an ethanol solution of MUA (mercapto alkanoic acid) solution with a concentration of 10 mM was used to incubate the sensing area at 40 °C for 30 min, in order to allow the MUA to attach to the gold film surface. Then, a 1:1 (volume ratio) mixed PBS (phosphate-buffered saline) solution of 0.4 mM EDC (1-ethyl-3-(3-dimethylaminopropyl) carbodiimide) and 0.1 mM NHS (N-hydroxysuccinimide) was used for mercapto activation. This procedure lasted for 30 min, and the pH of the mixed solution was 7.2. Then, the HER2 aptamer was immobilized onto the modified gold surface at room temperature for 30 min through the injection of PBS solution of the HER2 aptamer at a concentration of 100 μg/mL. Finally, to prevent non-specific adsorption in subsequent antigen–antibody binding tests, a blocking step was performed by modifying the sensing area in a 0.4 mM PBS solution of bovine serum protein (BSA) at room temperature for 10 min. It should be noted that PBS buffer was used to clean the modified gold surface for 10 min at the end of each step. The transmission spectrum’s evolution and the resonant wavelength variation of the proposed device during the bio-modification procedures are shown in Figure 4b,c, respectively. The abrupt change in resonant wavelength between adjacent steps was mainly caused by the RI differences in the solutions used successively. With these bio-modification procedures, the as-prepared polymer fiber SPR sensor was ready for HER2 detection.

## 3. Results and Discussion

**Figure 5:**
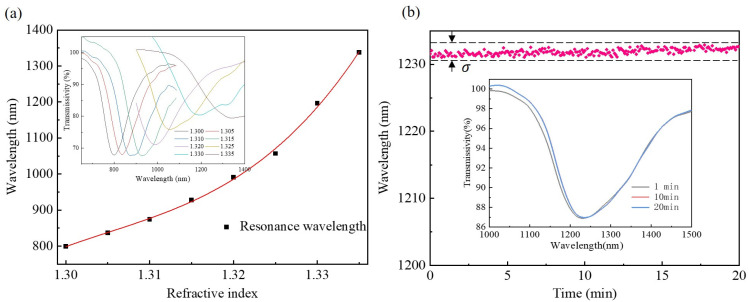
(**a**) SPR wavelength shift with surrounding RI increasing. (**b**) SPR wavelength fluctuation in PBS buffer for 20 min. Insets show the transmission spectrum’s evolution during the test.

The sensitivity of the D-shaped polymer fiber SPR device before bio-modification was evaluated by the use of standard RI liquids (Cargille Labs, Cedar Grove, NJ, USA), with the RI increasing from 1.300 to 1.335. The wavelength shift of the device corresponding to these analytes is shown in Figure 5a, where the inset demonstrates the transmission spectrum’s evolution with the surrounding RI increasing. Note that the spectra were collected by two independent spectrometers with operation ranges of 300–1100 nm and 900–1700 nm, respectively, due to the ultra-large wavelength shift of the proposed device. The resonant wavelength redshifted (shifted toward longer wavelengths) from 805 nm to 1325 nm nonlinearly, meaning that the sensitivity was accelerated and enhanced with the increase in the surrounding RI. The sensitivity reached 28,100 nm/RIU at around 1.330 of the surrounding RI, which is one order of magnitude higher than conventional silica fiber SPR sensors [35]. After bio-modification, the polymer fiber SPR sensor was immersed in a PBS solution for 20 min to verify its stability. The resonant wavelength fluctuation is plotted in Figure 5b, where one can see that the fluctuation in the resonant wavelength was less than 1.5 nm, as labeled between two dashed lines. According to these data, the standard deviation of the resonant wavelength could be calculated to be σ = 0.4338 nm. From the spectra shown in the inset of Figure 5b, the FWHM can be observed to be ~225 nm, apparently broader than that of the simulation results. This led to a degradation of FOM of ~124.9, considering a RI sensitivity of 28,100 nm/RIU. The broadening of FWHM was mainly caused by high-order-mode resonance-induced spectra deterioration of the multimode polymer fiber which was used.

**Figure 6:**
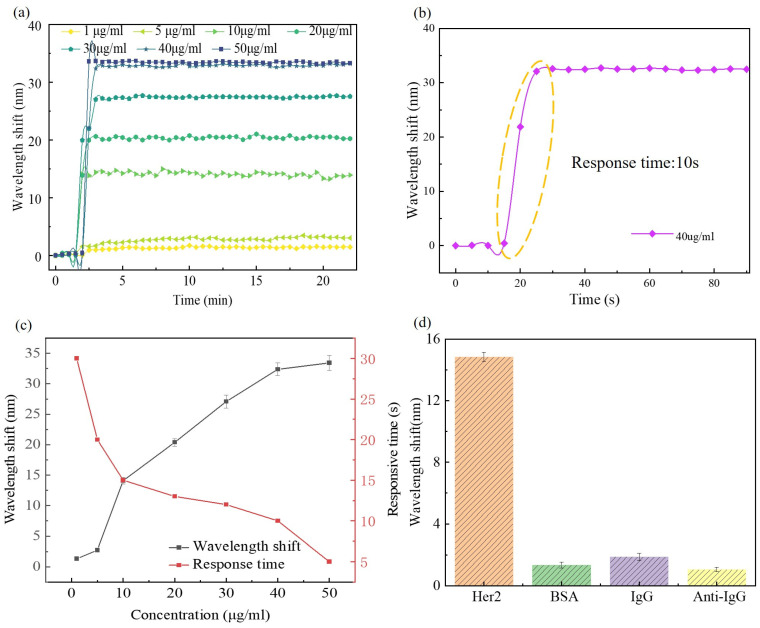
(**a**) SPR wavelength shift during HER2 detection for different concentrations. (**b**) Detailed SPR wavelength shift that demonstrates a response time of 10 s (estimated from the yellow circled data) for the detection of HER2 with a concentration of 40 μg/mL. (**c**) Wavelength shift and response time versus HER2 concentration. (**d**) Specific detection of HER2.

To evaluate the HER2 detection ability of the biosensor, PBS solutions of HER2 with concentrations of 1 μg/mL, 5 μg/mL, 10 μg/mL, 20 μg/mL, 30 μg/mL, 40 μg/mL, and 50 μg/mL were prepared and injected sequentially into the microfluidic channel as shown in Figure 1. Note that the biosensor was immersed in PBS solution initially and was cleaned with PBS buffer again at the end of HER2 detection for each concentration. The resonant wavelength of the biosensor was recorded every 1 min during the detection process, and the wavelength shift for each concentration of HER2 is plotted in Figure 6a. When the analyte flowed across the sensing area, the spectral shift was caused by the RI change in the analyte itself immediately, and then the specific combination between the antibody and the antigen increased the RI at the sensing surface, making the resonant wavelength further shift to longer wavelengths. The specific antigen–antibody binding generally took a few tens of seconds, resulting in a saturation of the wavelength shift of the biosensor for all cases of HER2 concentration. According to the total wavelength shift in the case of 1 μg/mL HER2 solution, the limit of detection (LOD) of the biosensor was estimated to be 0.95 μg/mL, namely, 5.28 nM, through the equation of LOD = 3σ/S, where σ is the standard deviation of the aforementioned resonant wavelength fluctuation and S is the total wavelength shift for a specific HER2 concentration of the biosensor. Typically, the response time is ~10 s for a HER2 concentration of 40 μg/mL, as depicted in Figure 6b. The total wavelength shift and corresponding response time are shown in Figure 6c for each concentration of HER2, where one can see clearly that the total wavelength shift increased while the response time decreased with the climbing HER2 concentration. Note that the response time was as fast as 5 s for a HER2 concentration of 50 μg/mL. Even for low concentrations, such as 1 μg/mL, the response time was still no longer than 30 s.

The specific detection of HER2 by the proposed biosensor was compared with the detection of BSA, human IgG, and anti-human IgG, respectively. Figure 6d presents the total wavelength shifts when bathing the biosensor in BSA solutions, 10 μg/mL human IgG solutions, and 10 μg/mL anti-human IgG solutions for one hour, respectively. The total wavelength shift for HER2 detection was ~14.85 nm, while those of BSA, human IgG, and anti-human IgG were only 1.34 nm, 1.88 nm, and 1.05 nm, respectively, implying that the surface-functionalized polymer fiber SPR biosensor can achieve a selective detection of HER2 with low cross-sensitivity to other agents.

The inclusion of an optical fiber biosensor into a small and portable device is important for practical and widespread clinical application. However, designing a D-shaped polymer optical fiber surface plasmon resonance biosensor for breast cancer detection applications presents several challenges, like sensitivity, selectivity, biocompatibility and stability, miniaturization, etc. We compared the performance of the proposed sensor with those of previously reported works, as listed in Table 1. To date, electrochemical-based sensors have made great progress, with the limit of detection (LOD) reaching the level of pg/mL. However, the proposed sensor exhibits advantages in terms of response time. Miniaturization of the sensor system is required for ease of use, cost-effectiveness, and scalability. Compared with traditional measurement methods, such as electrochemical, Biacore, and other methods, SPR fiber-optic sensors have the advantages of small size and fast response time. As shown in the table, the lower limits of different detection methods are different. However, based on the SPR sensor, a lower limit of detection was needed in this paper in order to reach the interval that could be applied, which has the advantages of fast measurement, device portability, etc. 

## 4. Conclusions

In this paper, we have demonstrated a highly sensitive D-shaped polymer optical fiber SPR biosensor for HER2 detection, which features the characteristics of portability, cost-effectiveness, and potential for early detection of breast cancer. The biosensor was fabricated by side-polishing a multimode polymer optical fiber with a length of 5 mm, a subsequent Au-film (thickness of 50 nm) coating, and HER2 antibody bio-modification. The biosensor exhibited an RI sensitivity of 28,100 nm/RIU, which is at least one order of magnitude higher than that of conventional silica fiber-based SPR sensors. For low-concentration detection of HER2, the biosensor exhibited a total wavelength shift of 1.37 nm with a HER2 concentration of 1 μg/mL, which corresponds to an LOD of 5.28 nM for HER2 detection. It should be noted that the response time of the proposed biosensor was measured to be as fast as 5 s for high concentrations of HER2. Even for low concentration tests down to 1 μg/mL (5.5 nM), the response time was still no longer than 30 s. The proposed biosensor exhibits good potential for the early detection of HER2 and offers a low-cost pathway to the early prevention of breast cancer. From the perspectives of portability and miniaturization, we expect to achieve miniaturization of the sensing system in the future. We will investigate the modularization of signal processing and miniaturization of the sensing system in future works.

## Figures and Tables

**Table 1:** Comparison of different test methods for HER2.

Measuring Method	LOD	Time	References
Reformative tyramine signal amplification	1.0 × 10^7^ particles/mL (3 pg/mL)	30 min	[36]
Aptasensors	15 ng/ml	-	[37]
Electrochemical affisensor	6.0 μg/l	60 min	[38]
Nanoelectrode ensembles	0.1 μg/ml	30 min	[39]
SPR-envelope biosensing	1 μg/ml	10 min	[40]
SPR fiber biosensing	0.95 μg/ml	10 s	

## Data Availability

Data are contained within the article.
